# Supplementary material for: Intrinsic surface p-wave superconductivity in layered AuSn4
Source: Nat Commun. 2023 Nov 2;14:7012. doi: 10.1038/s41467-023-42781-7 (PMC10622569; doi:10.1038/s41467-023-42781-7)
Supplement: Supplementary file 1 — Supplementary Information [file 41467_2023_42781_MOESM1_ESM.pdf]

Supplementary Information

Intrinsic Surface *p*-wave Superconductivity in Layered $AuSn_4$

Wenliang Zhu[1,#], Rui Song[2,#], Jierui Huang[3,#], Qi-Wei Wang[4,#], Yuan Cao[1,#], Runqing Zhai[1], Qi Bian[5], Zhibin Shao[1], Hongmei Jing[1], Lujun Zhu[1], Yuefei Hou[6], Yu-Hang Gao[4], Shaojian Li[5], Fawei Zheng[6], Ping Zhang[6,7*], Mojun Pan[3], Junde Liu[3], Gexing Qu[3], Yadong Gu[3], Hao Zhang[1], Qinxin Dong[3], Yifei Huang[3], Xiaoxia Yuan[8], Junbao He[9], Gang Li[3,10,11], Tian Qian[3,10,11,*], Genfu Chen[3,10,11,*], Shao-Chun Li[4,*], Minghu Pan[1,5,*] and Qi-Kun Xue[12,13,14,*]

[1]School of Physics and Information Technology, Shaanxi Normal University, Xi'an 710119, China

[2] Science and Technology on Surface Physics and Chemistry Laboratory, Mianyang 621908, China

[3]Institute of Physics and Beijing National Laboratory for Condensed Matter Physics, Chinese Academy of Sciences, Beijing 100190, China

[4]National Laboratory of Solid State Microstructures, School of Physics, Collaborative Innovation Center of Advanced Microstructures, Nanjing University, Nanjing 210093, China

[5]School of Physics, Huazhong University of Science and Technology, Wuhan 430074, China

[6]Institute of Applied Physics and Computational Mathematics, Beijing 100088, China

[7] School of Physics and Physical Engineering, Qufu Normal University, Qufu 273165, China

[8] Shaanxi Applied Physics and Chemistry Research Institute, Xi'an 710061, China.

[9] College of Physics and Electronic Engineering, Nanyang Normal University, Nanyang, 473061, China

[10]School of Physical Sciences, University of Chinese Academy of Sciences, Beijing 100190, China

[11]Songshan Lake Materials Laboratory, Dongguan, Guangdong 523808, China

[12]State Key Laboratory of Low-Dimensional Quantum Physics, Department of Physics, Tsinghua University, Beijing 100084, China

[13] Beijing Academy of Quantum Information Sciences, Beijing 100193, China

[14] Department of Physics, Southern University of Science and Technology, Shenzhen 518055, China

[#]These authors contribute equally to this work.

*Corresponding author. E-mail: zhang_ping@iapcm.ac.cn (P. Zhang), tqian@iphy.ac.cn (T. Qian), gfchen@iphy.ac.cn (G. Chen), scli@nju.edu.cn (S. Li), minghupan@snnu.edu.cn (M. Pan), qkxue@mail.tsinghua.edu.cn (Q. Xue).

Notes

Note 1. X-ray single crystal structural analysis....4
Note 2. Crystal structure and chemical composition....5
Note 3. Structural model and HAADF-STEM measurements....6
Note 4. The detailed information from the two-band fitting....7
Note 5. Anisotropic magnetotransport properties....7
Note 6. Point-contact Spectroscopy....8
Note 7. Surface topography, *dI/dV* spectroscopy and edge states....9
Note 8. CDWs at a half and full terrace edges....11
Note 9. Calculated band structures for $AuSn_4$....12
Note 10. Multiple Surface states and CECs measured at various energies....13
Note 11. The analysis of pairing symmetry of superconducting $AuSn_4$....14
Note 12. Two-fold symmetry induced by two-component SC at the surface of $AuSn_4$....15
Note 13. STS measured on the terrace and at the edge....20
Note 14. The correlations between SC gap and CDW modulations....21
Note 15. Two-fold angular dependence of the zero-resistance temperature....22
Note 16. Photon-energy-dependent ARPES measurements....24
Note 17. Band structure for the slabs and phonon spectrum....25
Note 18. The analysis of the size of the vortex lattice and the vortex core radius....27

Figures and Tables

Table S1. Sample and crystal data....4
Fig. S1. The single-crystal XRD of (00*l*) reflections and EDX patterns....5
Fig. S2. Ball-and-stick model and the simulated electron diffraction image. 6
Fig. S3. The angular dependence of the resistivity....7
Fig. S4. Temperature dependence of resistance for four-point probe setup....8
Fig. S5. Surface topography, *dI/dV* spectroscopy and edge states at 77 K....10
Fig. S6. CDW patterns at a half and full terrace edges....11
Fig. S7. Calculated band structures for bulk and monolayer $AuSn_4$....12
Fig. S8. Multiple Surface states near E_F....13
Fig. S9. CECs measured at various energies....13
Fig. S10. The angular dependence of the resistivity with different fittings....14
Fig. S11. Fermi surface of the normal state....15
Fig. S12. Mixture of *s*- and *p*-wave....17
Fig. S13. Splitting of orbits....18
Fig. S14. Multiple Rashba bands....19
Table S2. Classification of different pairings....19
Fig. S15. STS measured on the terrace and at the edge....20
Fig. S16. The correlations between SC gap and CDW modulations....21
Fig. S17. Schematic measurement configuration under in-plane magnetic fields....22
Fig. S18. Two-fold angular dependence of the zero-resistance temperature....23

Fig. S19. Photon-energy-dependent ARPES measurements..24
Fig. S20. Band structure for slabs with different thickness and phonon spectrum.....................26

References...28

Note 1. X-ray single crystal structural analysis

The structure of our $AuSn_4$ compound was determined by X-ray single crystal diffraction. The results are in below.

Table S1. Crystallographic data and details of the structure determination for $AuSn_4$.

**Parameter**	Value
**Chemical formula**	$AuSn_4$
**Formula weight (g/mol)**	671.73
**Temperature (K)**	293(2)
**Radiation wavelength (Å)**	0.71073
**Crystal size (mm³)**	0.946×0.951×0.126
**Crystal system**	orthorhombic
**Space group**	*Aba2* (No.41)
**Unit cell dimensions**	
a **(Å)/** ***α*** **(deg)**	6.476(2)/90°
b **(Å)/** ***β*** **(deg)**	6.476(2)/90°
c **(Å)/** ***γ*** **(deg)**	11.666(5)/90°
**Volume (Å³)**	497.97(19)
**Z**	4
**Calculated density (g/cm³)**	9.121
**Absorption coefficient (mm^{-1})**	49.831
**F (000)**	1116

Note 2. Crystal structure and chemical composition

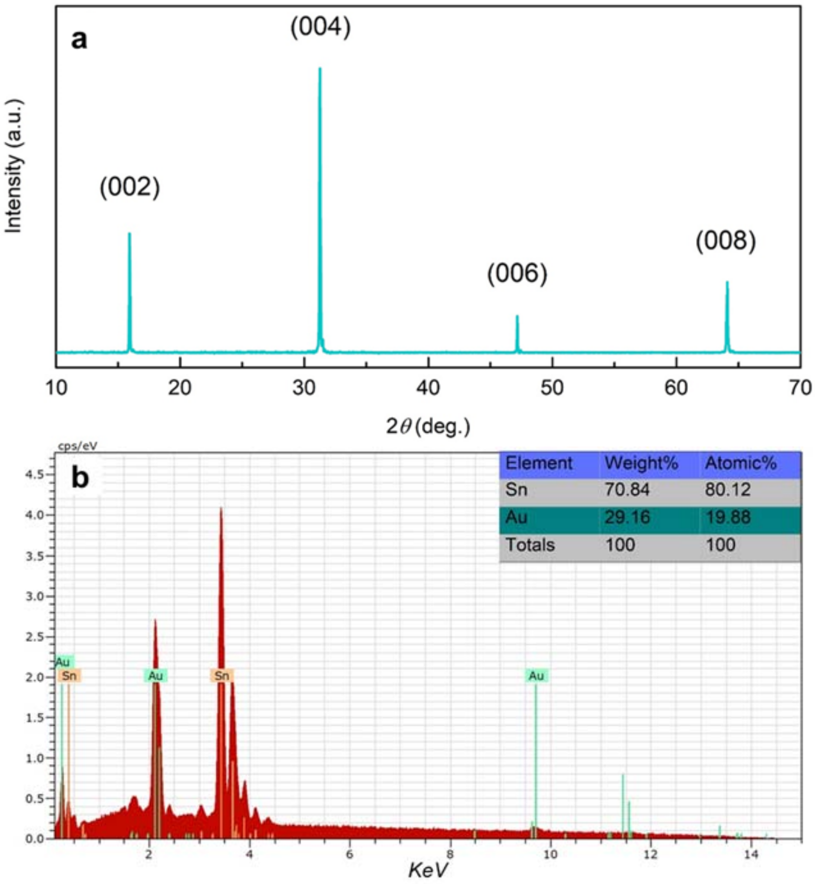

Supplementary Fig. S1 | a, The single-crystal XRD of (00*l*) reflections, indicating that the crystal grows well along the *ab* plane. **b**, EDX patterns of $AuSn_4$. The average Au : Sn atomic ratio determined using the EDX is very close to 1:4 and no foreign elements were detected within the limitation of instrument resolution.

Note 3. Structural model and HAADF-STEM measurements

Based on the data of X-ray single crystal diffraction, we construct the structural model for our $AuSn_4$ sample and performed atomic-level high-angle annular dark field scanning transmission electron microscopy (HAADF-STEM) imaging. As seen in Figs. S1a-1b, our $AuSn_4$ sample has A-B stacked Sn-Au-Sn trilayers, each Au layer is sandwiched by two Sn layers by forming a square lattice. The structure is in the space group *Aba2* with the lattice constants of *a, b* = 6.476 Å and *c* =11.666Å, which is significantly different from the reported structure with $PtSn_4$ and $PdSn_4$, which hold orthorhombic (*Ccca*, No. 68) space-group structure[1,2]. Further the measured and simulated electron diffraction image of $AuSn_4$ with space group *Aba2* and the simulated electron diffraction image of $AuSn_4$ with *Ccca* confirms this crystalline structure of our $AuSn_4$ sample, see Fig.1c and Figs. S1c-1d. The major difference between the structure of our $AuSn_4$ and the reported structure with $PtSn_4$ and $PdSn_4$, is the square-net Sn layer in our $AuSn_4$, which is also confirmed by our STM imaging.

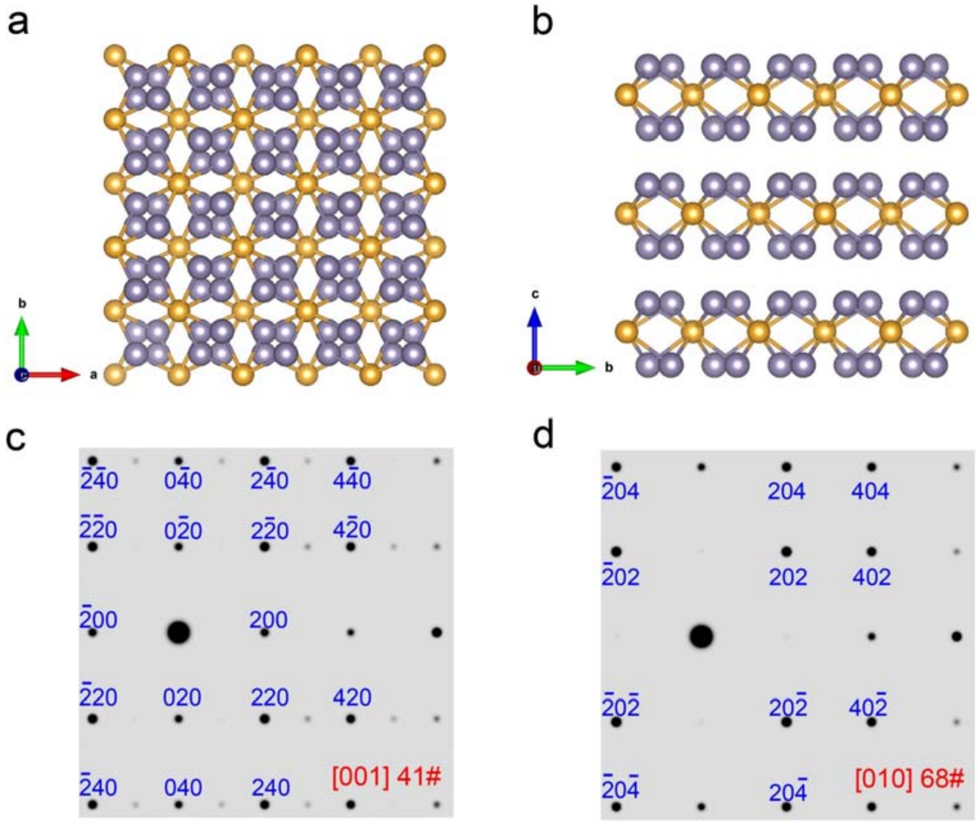

Supplementary Fig. S2 | a-b, The top and side views of ball-and-stick model for our $AuSn_4$ sample. **c-d,** The simulated electron diffraction image of $AuSn_4$ with space group *Aba2* No. 41 and *Ccca*, No. 68 along the *c* and *b* axis, respectively.

Note 4. The detailed information from the two-band fitting

A two-band superconductor can be described with the following equation[3]:

$$a_0[\ln t + U(t)][\ln t + U(\eta h)] + a_1[\ln t + U(h)] + a_2[\ln t + U(\eta h)] = 0,$$

where $a_0 = 2(\lambda_{11}\lambda_{22} - \lambda_{12}\lambda_{21})/\lambda_0$, $a_1 = 1 + (\lambda_{11} - \lambda_{22})/\lambda_0$, $a_2 = 1 - (\lambda_{11} - \lambda_{22})/\lambda_0$, $t = T/T_c$, $\eta = D_2/D_1$, $h = H_{c2}D_1/2\emptyset_0 T$, $\lambda_0 = \sqrt{(\lambda_{11} - \lambda_{22})^2 + 4\lambda_{12}\lambda_{21}}$, $\varpi = \lambda_{11}\lambda_{22} - \lambda_{12}\lambda_{21}$, and $U(x) = \psi(x+1/2) - \psi(1/2)$. $\psi(x)$ is the digamma function. D_1 and D_2 are the intraband diffusivities of each band. λ_{11} and λ_{22} are the intraband coupling constants. λ_{12} and λ_{21} are the interband coupling constants. Here, as the blue line shown in Fig. 1e, the best fitting parameters are D_1 =379.3704, D_2 =3.594, λ_{11} =0.0034, λ_{22} =0.1046, λ_{12} =0.0129 and λ_{21}=0.0321.

Note 5. Anisotropic magnetotransport properties

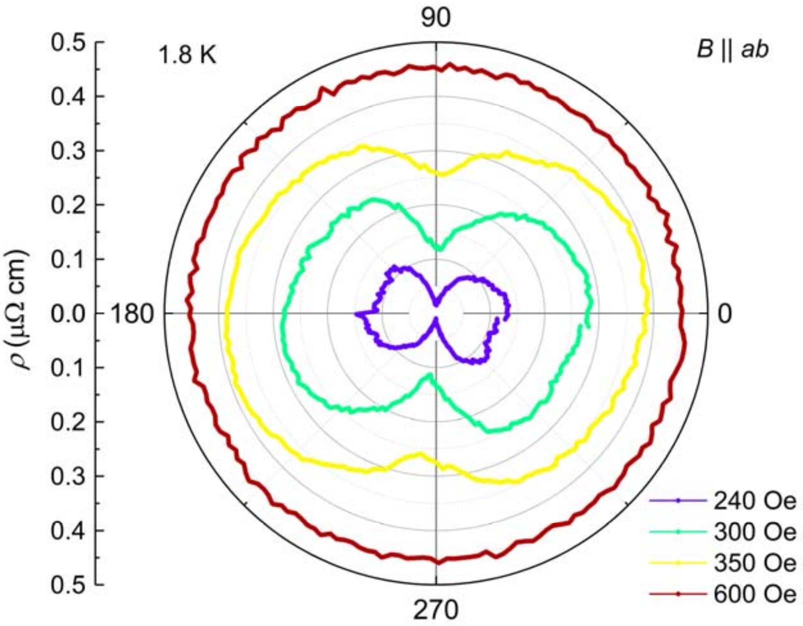

Supplementary Fig. S3 | The angular dependence of the resistivity at different magnetic fields and 1.8 K with the field rotating in the *ab*-plane.

Note 6. Point-contact Spectroscopy

The contact is made between a small drop (about 20-50 μm in diameter) of Ag paste and the surface of $AuSn_4$ single crystal, as shown in the inset of Fig. S4. The Ag electrode is connected to current and voltage leads through a thin Pt wire (18 μm in diameter) stretched over the sample. Differential *dI*/*dV* spectra are measured by the standard lock-in technique.

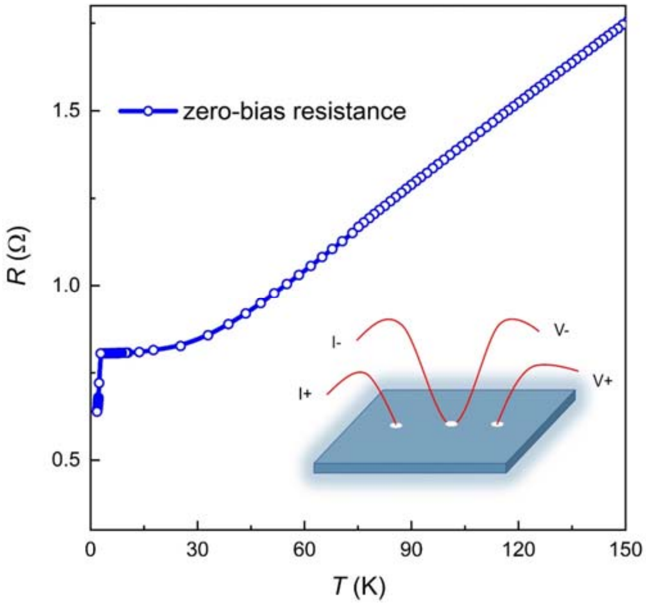

Supplementary Fig. S4 | Temperature dependence of the sample and junctions' resistance for the Ag-surface of $AuSn_4$ sample using the modified four-point probe setup. This distinct drop mainly contributed by the N/S contact resistance. Inset: Schematic diagram showing the soft point-contact on the Ag-surface of $AuSn_4$ sample and the differential conductance measurement electrodes.

Note 7. Surface topography, *dI/dV* spectroscopy and edge states

A large-scaled STM image (Fig. S5a) shows the clean (001) surface after ultrahigh vacuum cleavage with the terraces and the step height about 1.1 nm, in consistence with the lattice constant of the *c*-axis 11.666 Å determined by XRD data and close to 11.707 Å that reported for $AuSn_4$. A spectroscopic survey (color plot in lower panel of Fig. S5b) taken along yellow dashed line in upper panel of Fig. S5b, shows uniform distributed local density of states (LDOS) on the terraces and also weak DOS modulation induced by troughs. A color plot of *dI/dV* spectroscopic survey (lower panel of Fig. S5c) taken across a single step edge, reveals the existence of edge states. We present typical *dI/dV* spectra measured on the terrace (T, black) and the step edge (E, red) in Fig. S5d. The edge states appear inside of the CDW gap with the energy of 54 meV above E_F. Besides, the overall density of states at the step edge, are also greatly suppressed, which may attribute to the suppression of surface states at the edge. The surface states are observed and will be discussed in later ARPES measurements.

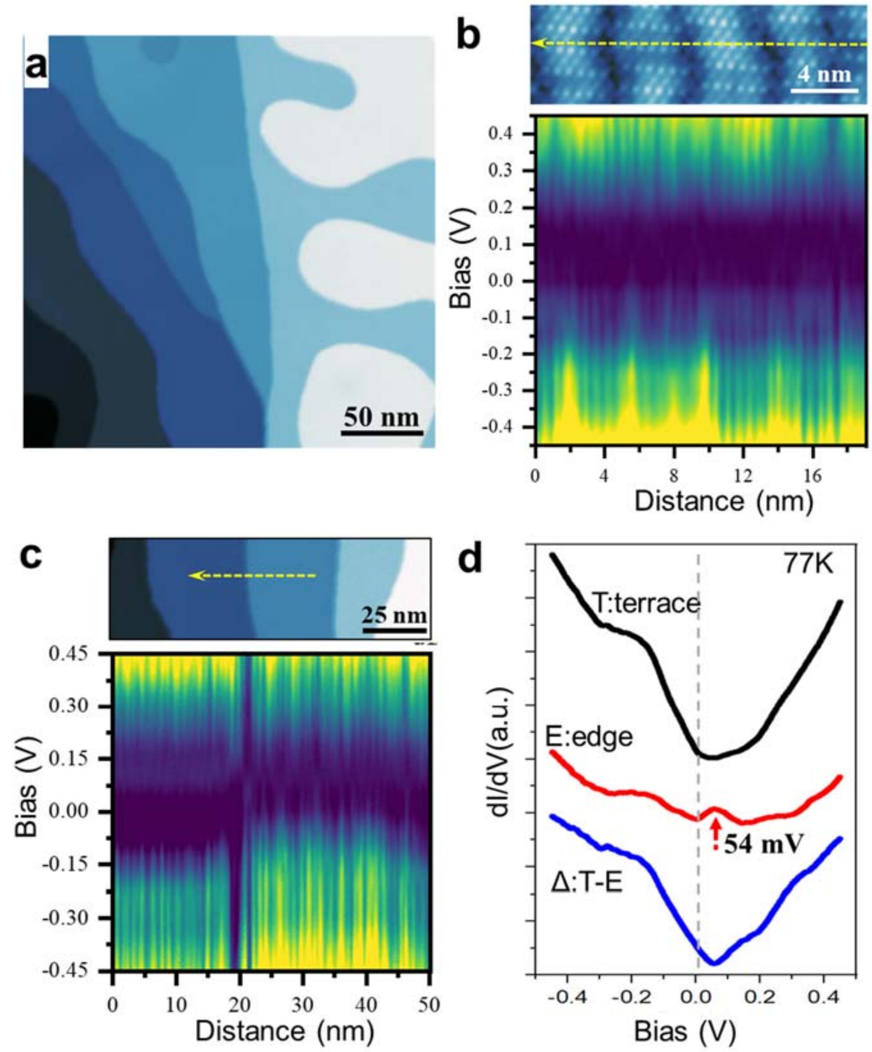

Supplementary Fig. S5 | Surface topography, *dI/dV* spectroscopy and edge states measured at 77 K. a, Large-scaled STM topographic image shows clean cleaved terraces with the step height about 1.1 nm, in consistence with the lattice constant of 11.666 Å along *c* axis. The image size is 250 × 250 nm^2. **b**, A spectroscopic survey taken along yellow dashed line in upper panel, shows the existence of 150 meV gap and weak DOS modulation induced by troughs. **c**, A color plot of *dI/dV* spectroscopic survey taken along yellow dashed line across a single step edge, reveals the existence of edge states. **d**, The representative *dI/dV* spectra measured on the terrace (T, black) and the step edge (E, red). The edge states appear inside of the CDW gap with the energy of 54 meV. The *dI/dV* spectra are taken with the setting parameters of V_B = 200 mV, I_T = 300 pA and standard lock-in technique with the modulation of 5.5 mV and the frequency of 973.0 Hz.

Note 8. CDWs at a half and full terrace edges

The $\sqrt{2}\times 2\sqrt{2}$ CDW phase and Q1D modulations may lead to anisotropic gap function, resembling to $Cu_xBi_2Se_3$[4]. However, STM images show the directions of CDW and Q1D modulations can be along either *a* or *b* axis, which will not introduce the two-fold anisotropic SC in macroscopic scale. As shown in Fig. S6, the full edge is with step height ~ 11 Å and the direction of CDWs (defined as the direction of $\sqrt{2}\times 2\sqrt{2}$) in the upper and lower terraces are perpendicular to each other (marked as red dashed arrows). The half edge is with step height ~ 5.6 Å and the direction of CDWs in the upper and lower terraces are parallel to each other (marked as red dashed arrows). All these results suggest that CDWs can be developed along both *a* and *c* directions, which cannot provide for the underlying mechanism of two-fold symmetry of SC.

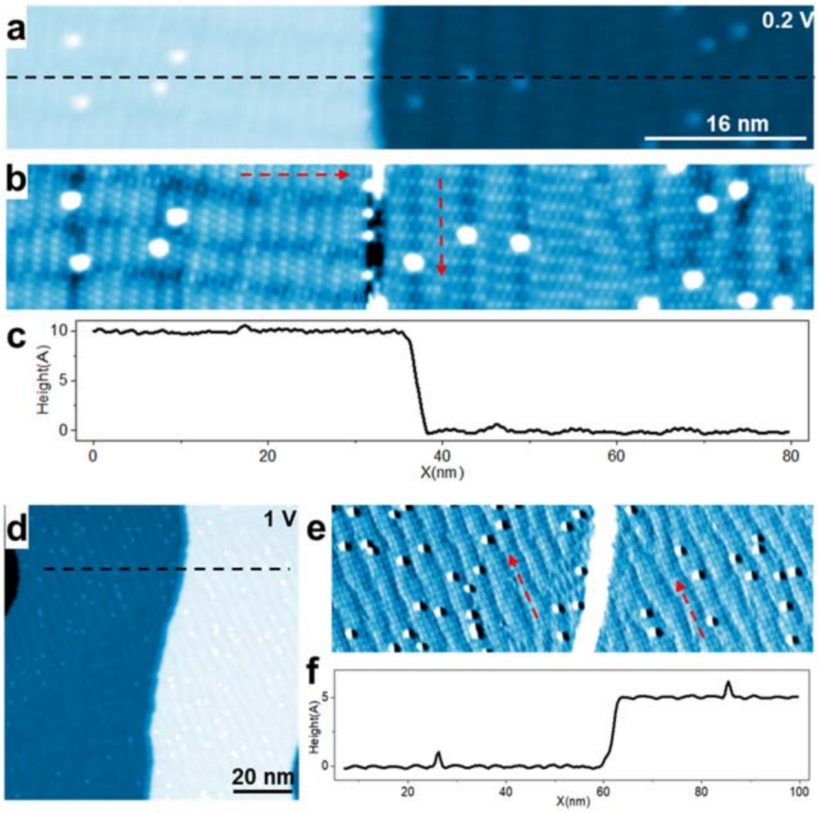

Supplementary Fig. S6 | a-c, topographic STM image, flatten STM image, height profile of terrace step for a full terrace edge, respectively. **d-f,** topographic STM image, flatten STM image and height profile of terrace step for a full terrace edge, respectively.

Note 9. Calculated band structures for $AuSn_4$

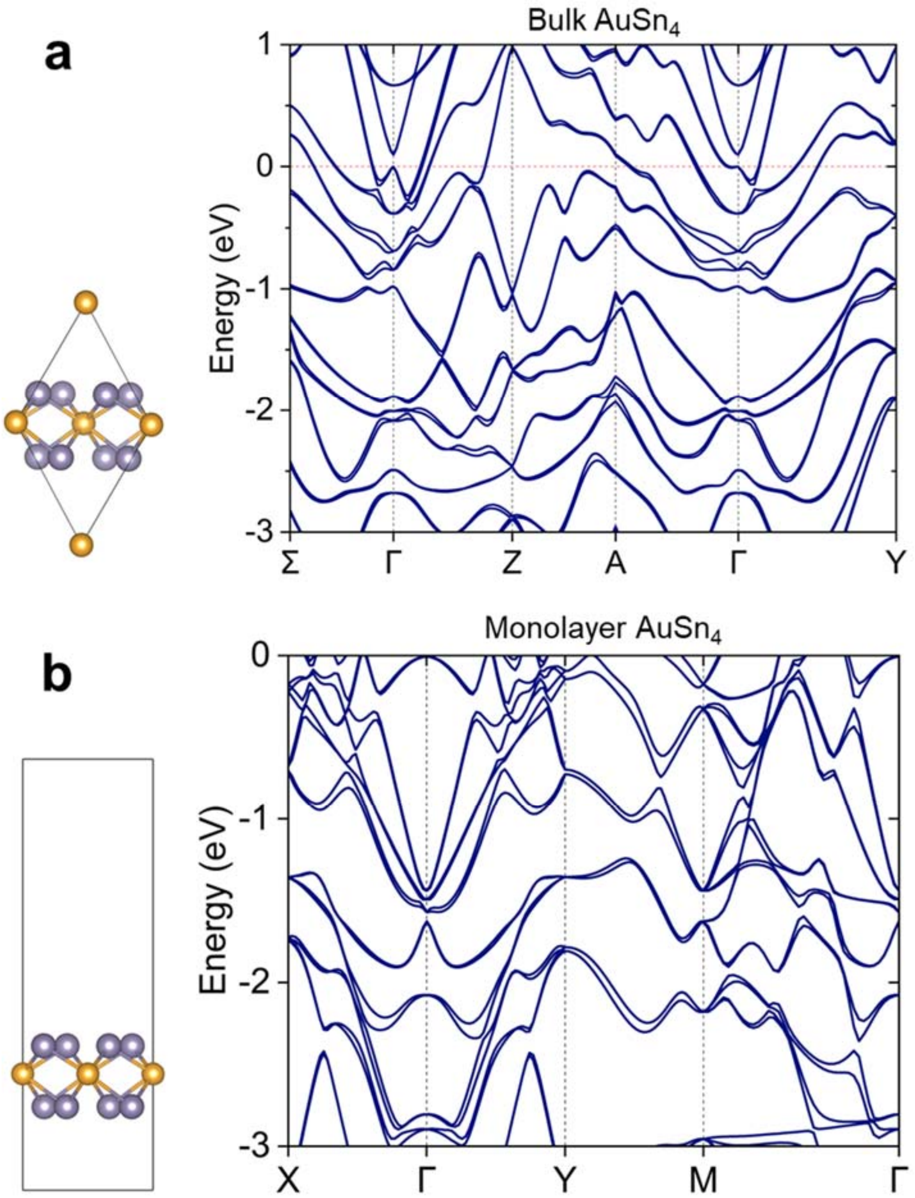

Supplementary Fig. S7 | Calculated band structures for $AuSn_4$ with considering bulk unit cell (**a**) and the monolayer $AuSn_4$ (**b**).

Note 10. Multiple Surface states and CECs measured at various energies

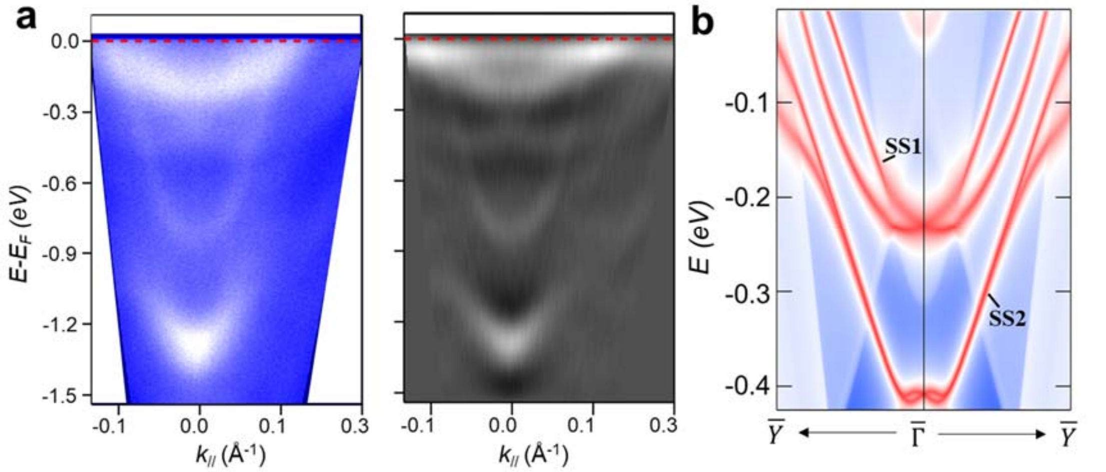

Supplementary Fig. S8 | Multiple surface states near E_F. a, (Left) Small energy-scaled band dispersion measured along Y-Γ-Y direction, where multiple surface bands are clear observed. (Right) the derivative plot of band dispersion. **b**, The calculated surface states from the bulk structure along Y-Γ-Y direction.

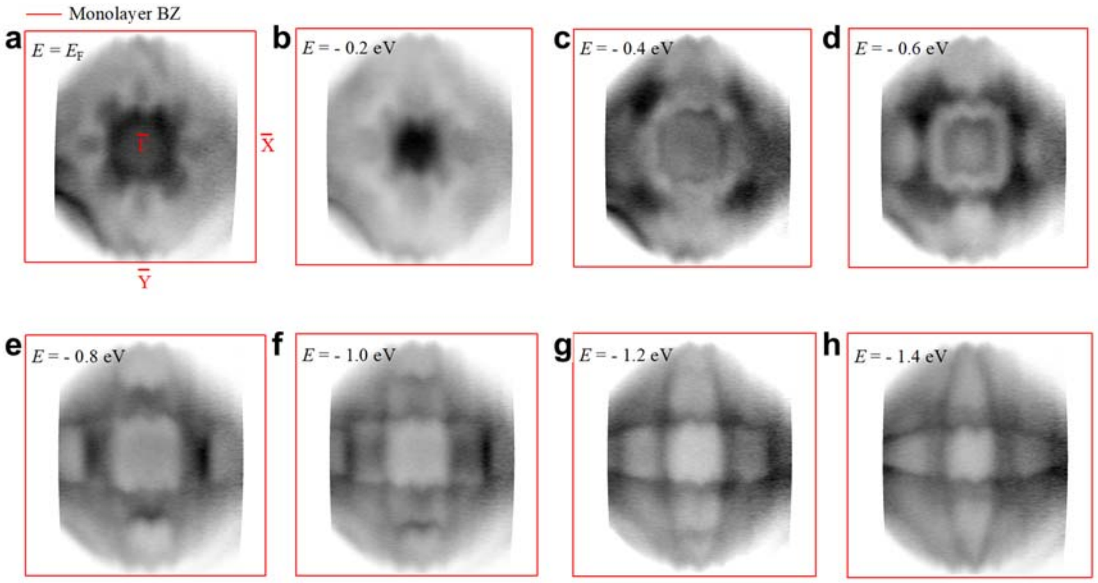

Supplementary Fig. S9 | A series of CECs measured at various energies from 0 to -1.4 eV.

Note 11. The analysis of pairing symmetry of superconducting $AuSn_4$

Anisotropic s wave and s + p wave pairing

We thus check the data of angular dependence of the resistivity at different magnetic fields (Fig. 2c) and replot it into Fig. S10a-10b. We find the anisotropic *s*-wave gap function could not fit the data well. These data can be fitted well by assuming the gap function of $\Delta = \Delta_0 + \Delta_1 cos^2\theta$, where the first term is *s*-wave gap, the second term is in *p*-wave symmetry, the overall shows the two-fold symmetry originated from the mixing of *s*- and *p*-wave parings.

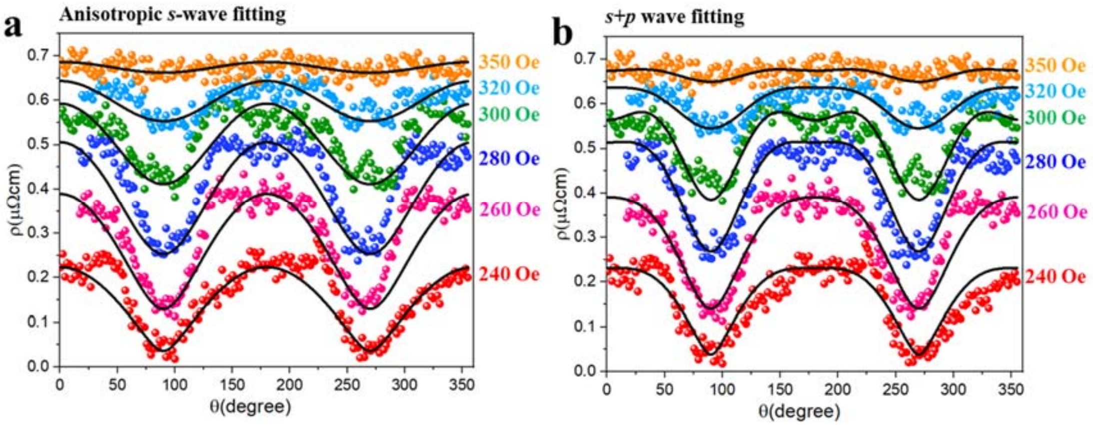

Supplementary Fig. S10 | a, The replotted angular dependence of the resistivity with different magnetic fields and the fitting based on an anisotropic s-gap function $\Delta = \Delta_1 cos^2\theta$. **b**, The replotted data and the fitting based on an *s*+*p* pairing gap function, $\Delta = \Delta_0 + \Delta_1 cos^2\theta$.

Note 12. Two-fold symmetry induced by two-component superconductivity at the surface of $AuSn_4$

12.1 Landau phenomenological theory

The ARPES experimental results show that the electronic structure at the surface of $AuSn_4$ is mainly determined by the topmost monolayer whose space symmetry can be described by point group C_{4v}. Due to the breaking of inversion symmetry, a typical Rashba band splitting would emerge in its band structure and can be described by the Hamiltonian:

$$H_R = t(cosk_x + cosk_y) + \alpha_R(sink_x\sigma_y - sink_y\sigma_x) - \mu \quad (1)$$

Obviously, the normal state opposes a four-fold rotational symmetry which is reflected in its Fermi surface as shown in Fig. S12, and that has also been verified by our ARPES experiment.

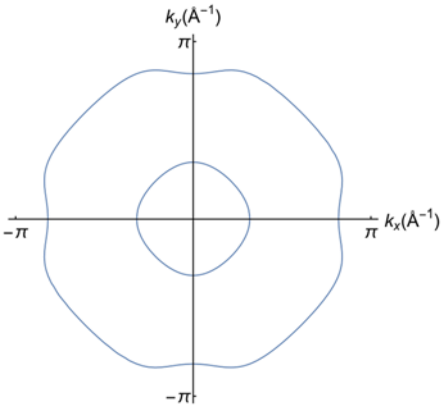

Supplementary Fig. S11 | Fermi surface of the normal state

When the system enters superconductivity, the symmetry is determined by the superconductivity order parameter $\Delta(\boldsymbol{k})$, whose performance is constrained by the point group and can be described by the Landau theory. In detail, there are five irreducible representations (IR) of C_{4v}, among which four are one-dimensional (A_1, A_2, B_1, B_2) and one is two-dimensional (E). The superconductivity should happen in one certain IR channel, and only the multi-dimensional channel allows multi-component superconductivity exists and consequently breaks the rotation symmetry. The order parameter is a linear combination of different components, which is

$$\Delta(\boldsymbol{k}) = \sum_m \eta(\Gamma, m)\hat{\Delta}(\Gamma, m; k). \quad (2)$$

Here, $\eta(\Gamma, m)$ are complex numbers. The stable superconducting state that orders according to the E representation would then be characterized by

$$\Delta_E(\boldsymbol{k}) = \eta_1(\Delta_{xz}(\boldsymbol{k}) + \boldsymbol{d}_1(\boldsymbol{k}) \cdot \boldsymbol{\sigma})i\sigma_y + \eta_2\big(\Delta_{yz}(\boldsymbol{k}) + \boldsymbol{d}_2(\boldsymbol{k}) \cdot \boldsymbol{\sigma}\big)i\sigma_y \quad (3)$$

where $\Delta_{xz/yz}(\boldsymbol{k}) \propto k_x k_z (k_y k_z)$ is singlet (odd parity) and $\boldsymbol{d}_1(\boldsymbol{k}) = k_x\hat{z}\ or\ k_z\hat{x},\ \boldsymbol{d}_2(\boldsymbol{k}) = k_y\hat{z}\ or\ k_z\hat{y}$ is triplet (odd parity)[5]. Eq. (3) also reflects the mixture of even and odd parity due to the breaking of inversion symmetry. Up to fourth order, the Landau free energy can be expanded by the complex order parameter as[5]:

$$F = A(T - T_c)(|\eta_1|^2 + |\eta_2|^2) + \beta_1(|\eta_1|^2 + |\eta_2|^2)^2 + \beta_2(\eta_1^*\eta_2 - \eta_1\eta_2^*)^2 + \beta_3|\eta_1|^2|\eta_2|^2 \quad (4)$$

When $T < T_c$, the minimum of the free energy determines the symmetry of the stable superconducting state. For $\beta_2 > 0$, the nematic superconductivity, given by $(\eta_1, \eta_2) = \eta_0(cos\theta, sin\theta)$ is favored. Here, θ is the nematic angle, which can be further determined by considering the sixth order in Landau free energy. It is easy to get that the nematic angle is pinned at *a* or *b* axis of the crystal, i.e. $(\eta_1, \eta_2) = (1,0)$ or $(0,1)$.

At the limitation of two dimension, it is naturally to take an approximation as $k_z = 0$, and then $\Delta_E(\boldsymbol{k})$ is reduced to a node p-wave superconducting gap as shown in Fig. S13(b). Note that this can only happen at the surface, because in the bulk, the superconducting symmetry is constrained by D_{2h} point group which describes the space structure of bulk $AuSn_4$. Since D_{2h} has no multi-dimensional IR, isotropy *s*-wave superconductivity is thus favored in the bulk. While the proximity effect can naturally mix the two different kinds of superconductivity, the total pairing potential thus can be expressed as:

$$\Delta(\boldsymbol{k}) = i\Delta_s\sigma_y + i\Delta_p(\eta_1 k_x\sigma_z + \eta_2 k_y\sigma_z)\sigma_y \quad (5)$$

where Δ_s and Δ_p is the amplitude of *s* and *p*-wave superconducting component. The transport measurement will inevitably reflect the property of it. As shown in Fig. S12, the mixture of surface and bulk superconductivity leads to a two-fold symmetry superconductivity.

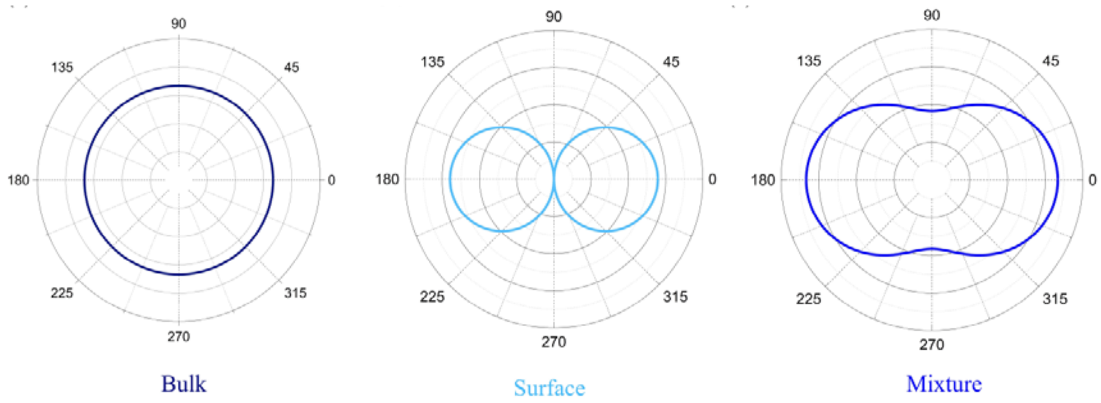

Supplementary Fig. S12 | Mixture of *s* and *p* wave, the nematic order is chosen as (η_1; η_2) = (1; 0).

12.2 Microscopic theory

As shown in our ARPES experimental results, the Fermi level crosses two Rashba-like surface states which are labelled as SS1 and SS2, thus the superconductivity can be seen as a Cooper condensation from these two bands. A typical Rashba band structure can be well described by Eq. (1), or more briefly, in a $k \cdot p$ approximation as

$$H_R = \varepsilon k^2 + \alpha_R(k_x\sigma_y - k_y\sigma_x) - \mu \quad (6)$$

However, when there exist two Rashba bands and close to each other in momentum space, the hybridization between them then can't be avoided. This process can be described by a total Hamiltonian which involves both the primitive two Rashba bands and their interactions, which is

$$H = H_R \otimes \tau_0 + H_{int} \quad (7)$$

The form of H_{int} is constrained by the symmetry of the system. Back to the actual-material environment, topmost monolayer $AuSn_4$, the dominant orbits will split under crystal field (CF) and spin-orbit coupling (SOC) as shown in Fig. S13. It is easy to derive that every Γ_6 IR can form a single Rashba band whose Hamiltonian is Eq. 6 at low-energy limitation. Usually *s* and p_z orbits have the close energy and thus hybridization happens, and p_x and p_y are degenerated[6]. Based on this, we can define two pseudo orbits, sp_z and p_xp_y (for simplicity, labelled as 1 and 2), both of them have the similar symmetry and can independently form a single Rashba band.

However, further hybridization between sp_z and p_xp_y are allowed because they belong to the same IR. Under the basis $\psi = (c_{1\uparrow}, c_{1\downarrow}, c_{2\uparrow}, c_{2\downarrow})^T$, the specific form of the total Hamiltonian can be expressed as :

$$H_0 = \begin{pmatrix} \varepsilon k^2 + \epsilon_0 & i\alpha_R k_- & 0 & \beta k_+ \\ -i\alpha_R k_+ & \varepsilon k^2 + \epsilon_0 & \beta k_- & 0 \\ 0 & \beta k_- & \varepsilon k^2 & i\alpha_R k_- \\ \beta k_+ & 0 & -i\alpha_R k_+ & \varepsilon k^2 \end{pmatrix} \quad (8)$$

It's obviously that, β term measures the coupling strength between two Rashba bands, when $\beta = 0$ it's a trivial system where two single Rashba bands have an energy difference ϵ_0.

As shown in Fig. S14a, it's a simple superposition of two Rashba bands when hybridization is absent. The coupling then would open a gap and introduce a band kink as shown in Fig. S14b, as a result, the Fermi surface gains an unconventional spin texture which profoundly changes the spin-transport property of the system[7]. Meanwhile, unconventional superconductivity may also emerge.

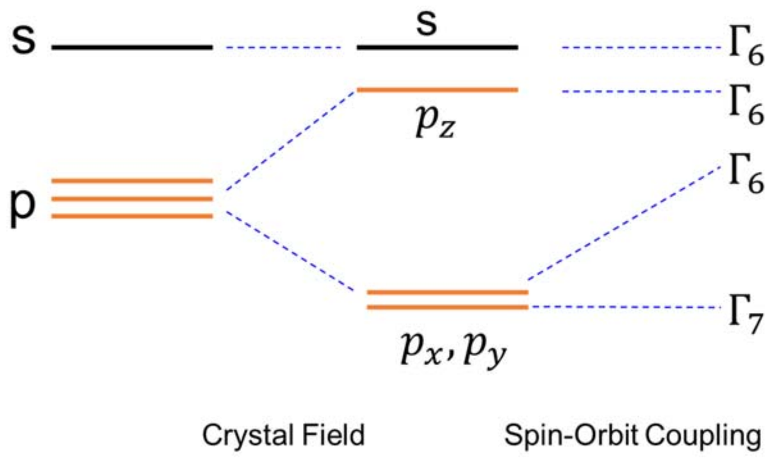

Supplementary Fig. S13 | Splitting of orbits.

Now, considering the system enters superconductivity, we can analyze the possible pairing mode. Based on the regular basis $\Psi = (\psi, \psi^\dagger)$, the superconducting order parameter obeys $\Delta^T = -\Delta$. For momentum-independent case, there are six possible pairing ways, which are $\delta_1 = \sigma_0 s_y$, $\delta_2 = \sigma_x s_y$, $\delta_3 = \sigma_z s_y$, $\delta_4 = \sigma_y s_0$, $\delta_5 = \sigma_y s_x$, $\delta_6 = \sigma_y s_z$. According to their symmetry, they can be classified as Table S2.

The inter-orbital triplet pairing belonging to the *E-IR* gives a two-component nematic superconductivity, combined with our experimental results, this is the most likely condition happening in the surface of $AuSn_4$.

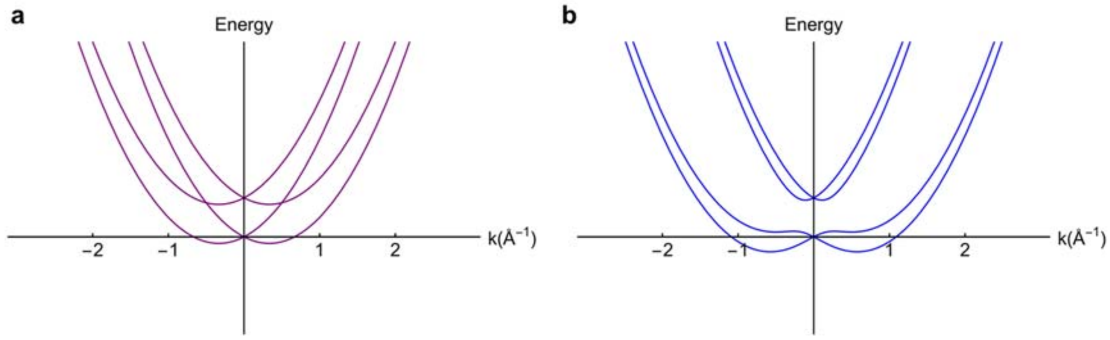

Supplementary Fig. S14 | Multiple Rashba bands (**a**) without and (**b**) with hybridization, respectively.

Order Parameter	Matrix form	Explicit form	IR
Δ_1	δ_1	$c_{1\uparrow}c_{1\downarrow} + c_{2\uparrow}c_{2\downarrow}$	A_1
	δ_2	$c_{1\uparrow}c_{2\downarrow} + c_{2\uparrow}c_{1\downarrow}$	
	δ_3	$c_{1\uparrow}c_{1\downarrow} - c_{2\uparrow}c_{2\downarrow}$	
Δ_2	δ_5	$c_{1\uparrow}c_{2\downarrow} - c_{2\uparrow}c_{1\downarrow}$	A_2
Δ_3	$\{\delta_4, \delta_6\}$	$\{c_{1\uparrow}c_{2\uparrow}, c_{1\downarrow}c_{2\downarrow}\}$	E

Supplementary Table. S2 | Classification of different pairings.

In summary, the unconventional superconductivity in $AuSn_4$ can be understood as the following: Firstly, the electronic structures of surface and bulk are distinct, especially the surface band structure are mainly determined by the topmost monolayer $AuSn_4$. Secondly, two coupled Rashba-type surface bands induce a special Fermi surface which opposes an unconventional spin texture, and that can add the unconventional pairing. Finally, the inter-orbit triplet pairing is induced in *E-IR* channel, which spontaneously breaks the rotational symmetry. Note that, the pure surface triplet superconductivity has nodes, only when mixed with the bulk full-gap isotropic *s*-wave superconductivity it becomes full-gap and two-fold symmetry.

Note 13. STS measured on the terrace and at the edge.

We performed the STS measurements on the flat terrace and at the edge at 160 mK. In Fig. S15, the SC gap measured at the edge (blue) shows a slightly smaller gap size and shallower gap depth, comparing to the gap measured on the flat terrace (red), both suggesting the possible existence of dispersive Majorana edge mode at the edge.

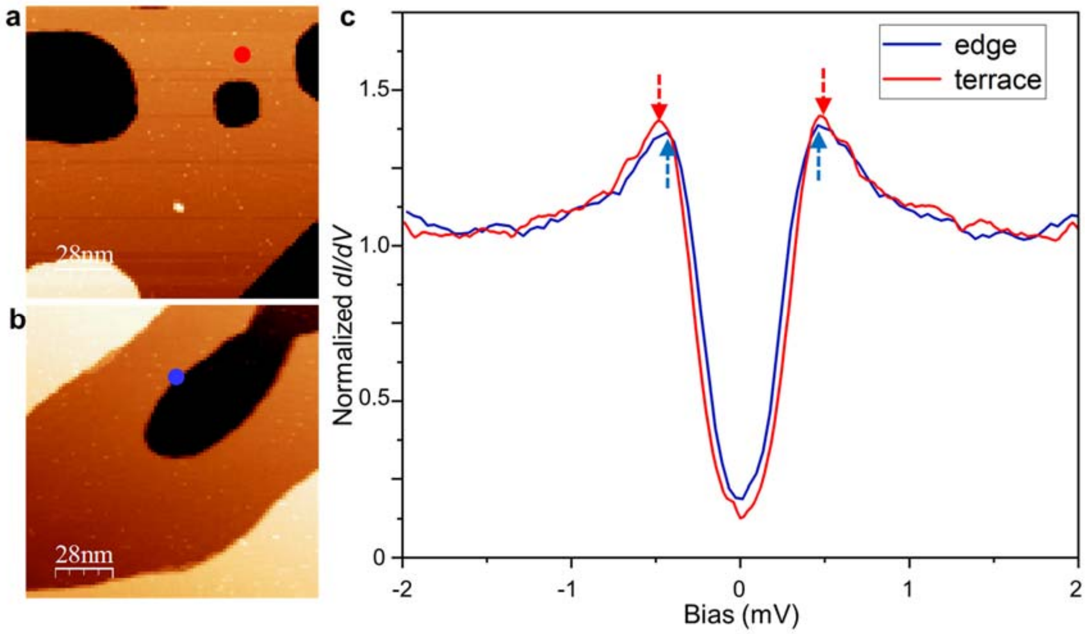

Supplementary Fig. S15 | STS measured on the terrace and at the edge. a-b, Topographic images showing the locations of point dI/dV spectra (**a**: I_t: 100 pA, V_b: 100 mV, and image size: 130×130 nm^2, **b**: I_t: 100 pA, V_b: 0.675 V, and image size: 130×130 nm^2). **c**, the comparison of *dI/dV* spectra on the terrace (red) and at the edge (blue). The STS set-up conditions: edge: $V_b = 6.75$ mV, $I_t = 100$ pA, $V_{mod} = 67.5$ µV, terrace: $V_b = 1.35$ mV, $I_t = 300$ pA, $V_{mod} = 27$ µV, taken at the temperature of 160 mK and magnetic field of 20 Oe.

Note 14. The correlations between SC gap and CDW modulations.

For the relationship between the superlattice and paring symmetry, we carried out STS measurements at 160 mK. As we mentioned, there are two different modulations on this surface, $\sqrt{2}\times2\sqrt{2}$ superstructure (CDW) and stripe-like pattern. As shown in Fig. S16, both line STS surveys, show clearly the SC gap modulations with both $\sqrt{2}\times2\sqrt{2}$ superstructure (CDW) and stripe pattern. Our new results suggest that the observed CDW state is relevant to the superconductivity, which may point out the nature of the coexisting triplet superconductivity and PDWs.

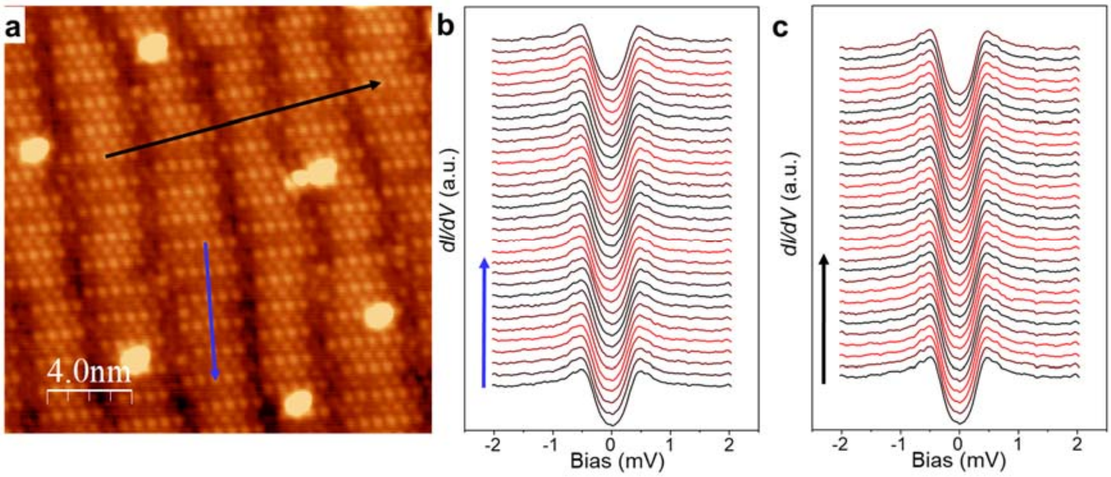

Supplementary Fig. S16 | The correlations between SC gap and CDW modulations. a, Topographic image (I_t: 100 pA, V_b: 1.0 V, and image size: 20×20 nm^2) taken at temperature of 4.2K. **b-c**, the line *dI*/*dV* spectroscopic survey taken along the blue (black) arrows in panel **a**. Inset: intensity map around the coherence peak energy, clearly showing the modulation of the coherence peaks in energy. The STS set-up conditions: **b**: $V_b = 2.7$ mV, $I_t = 200$ pA, $V_{mod} = 40$ μV, **c**: $V_b = 1.35$ mV, $I_t = 200$ pA, $V_{mod} = 27$ μV taken at the temperature of 160 mK.

Note 15. Two-fold angular dependence of the zero-resistance temperature.

In order to exclude extrinsic factors, we performed the angle-dependent magnetoresistance measurements with the current *I* along *a* and *b* axis, as shown in Fig. S17a and 17b, respectively. Here, θ is the angle between the directions of fields *B* and current *I*, not the angle between the directions of fields *B* and *a* axis. When $\theta = 0°$, it is worth to note that the current *I* is parallel to the *b* axis in Fig. R17a and the current *I* is parallel to the *a* axis in Fig. R17b.

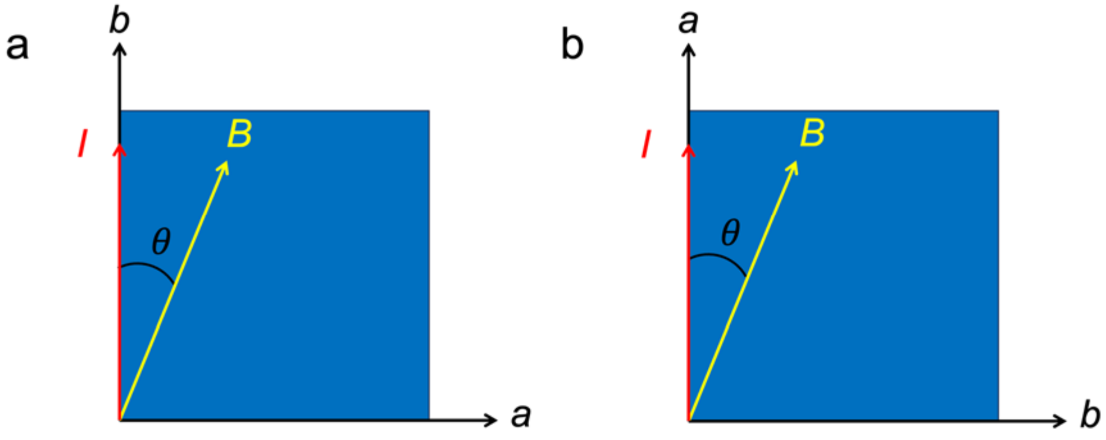

Supplementary Fig. S17 | Schematic measurement configuration under in-plane magnetic fields. a and **b**, the current *I* is parallel to *b* and *a* axis, respectively. θ is defined as the angle between the fields *B* and current *I*.

We provide the temperature-dependent resistivity under a given in-plane field 120 Oe with the current *I* along *a* and *b* axis, as shown in Figs. S18a-c and Figs. S18d-e, respectively. Here, the zero-resistance temperature (T_c^0) is defined as the intersection of the tangent line and the *R*-*T* curve, indicated by the thin arrow. Figure S18f shows the angle dependence of the zero-resistance temperature extracted from Figs. S18b-e. We find that the two angular dependences in Fig. S18f are not identical, consistent with the results of the angle-dependent magnetoresistance in the range between the onset and the offset of superconductivity.

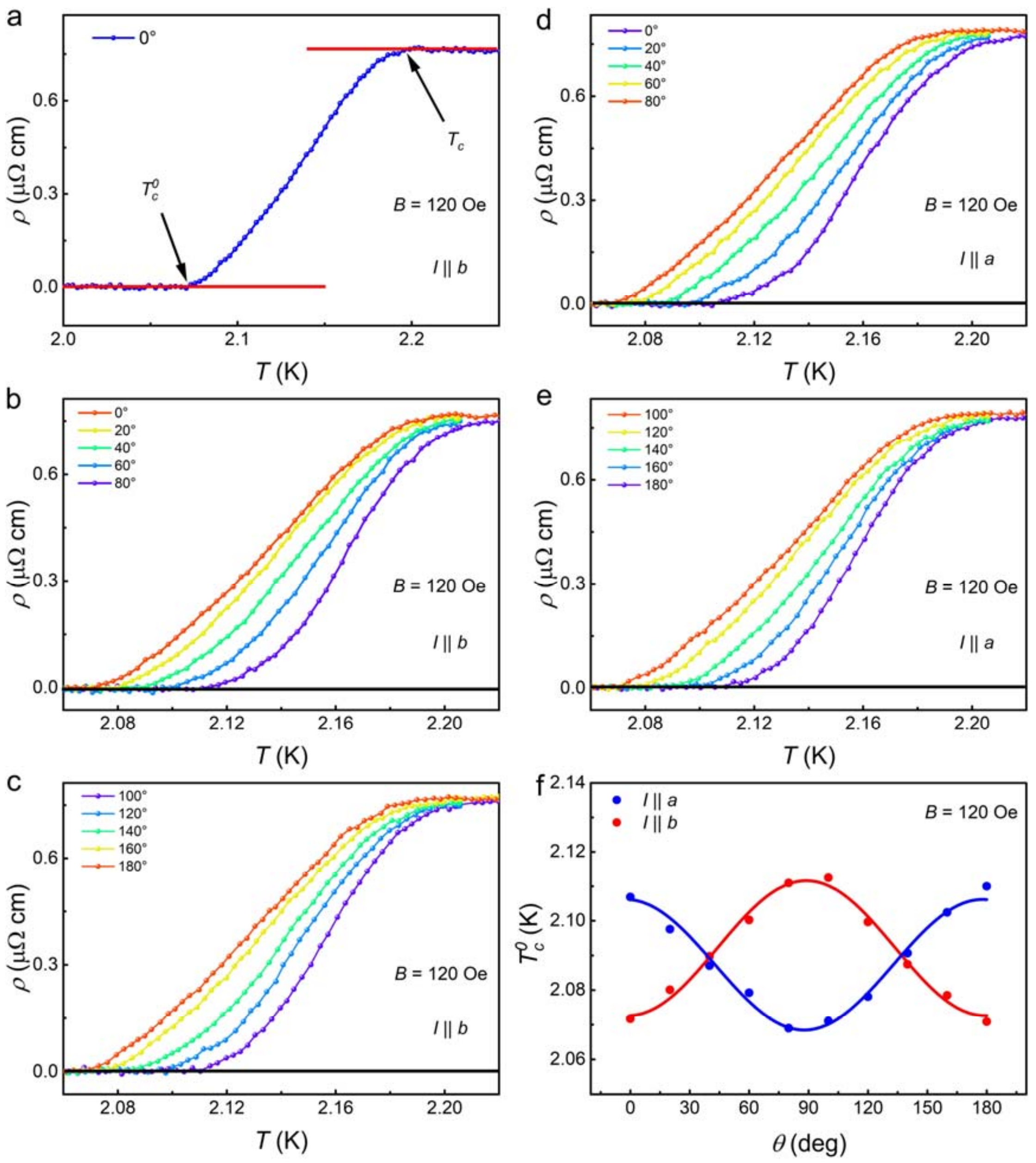

Supplementary Fig. S18 | Two-fold angular dependence of the zero-resistance temperature of superconducting $AuSn_4$ under a given in-plane magnetic fields. a-c, The temperature-dependent resistivity with the current *I* along *b* axis under 120 Oe and selected angel. **d-e**, The temperature-dependent resistivity with the current *I* along *a* axis under 120 Oe and selected angel. **f**, The angle-dependent T_c^0 extracted from the panels of **b-e**.

Note 16. Photon-energy-dependent ARPES measurements

We carry out photon-energy-dependent ARPES measurements at the Dreamline beamline of the Shanghai Synchrotron Radiation Facility with a Scienta Omicron DA30L analyzer. We show the data of k_z dispersion in Fig. S19, in which photon-energy-dependent band dispersion is almost a series of vertical lines, suggesting that there is no obvious k_z dispersion and the coupling between two single layers is very weak. So the motion of electrons in $AuSn_4$ is restricted in each 2D single layer, especially for the surface electrons. Besides, we find that the photon energy of about 22 eV is a good choice for ARPES studies of $AuSn_4$.

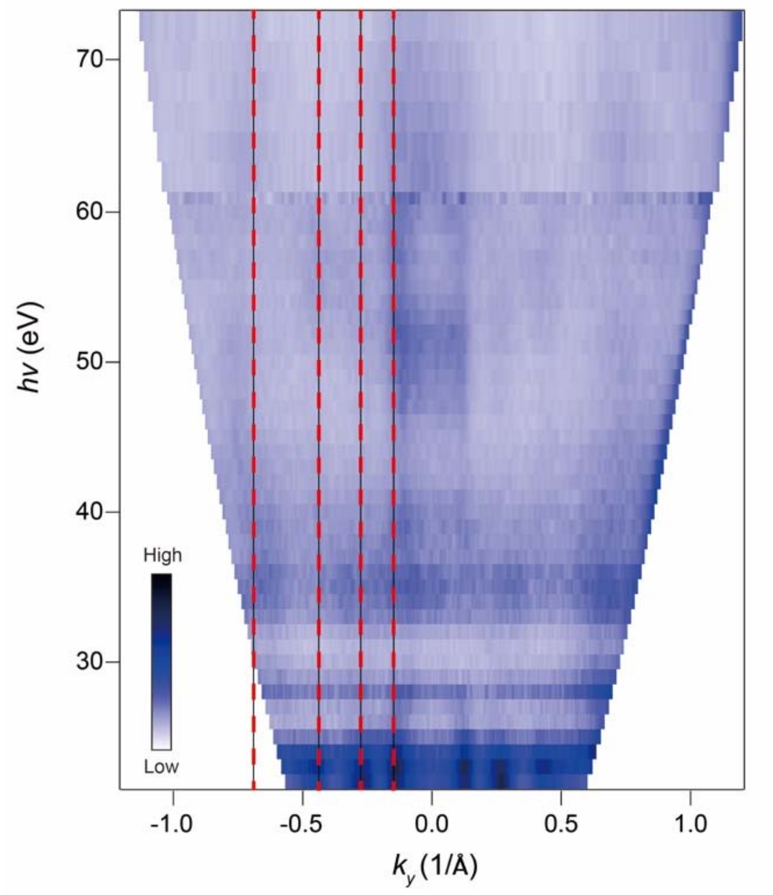

Supplementary Fig. S19 | Photon-energy-dependent ARPES measurements show that there is no obvious k_z dispersion.

Note 17. Band structure for the slabs and phonon spectrum.

As shown in Fig. S20, different slab models are adopted in order to figure out the origination of the ARPES-measured bands. The corresponding band structure shows a good metallicity, however, none of them agrees with the ARPES-measured results. $2\sqrt{2}\times\sqrt{2}$ supercell slab and the corresponding band structure is shown in Fig. S20d. Obviously, it deviates the ARPES measurements a lot, which indicates that the ARPES results cannot be interpreted by surface superstructure. The phonon band of bulk $AuSn_4$ shown in Fig. S20f and S20g indicates an evident dynamic stability of this system, which demonstrate that the superstructure on the surface (CDW-like state) is not originated from crystal lattice instability. In conclusion, the slab model cannot capture the dispersion relationship nor the Fermi surface's symmetry detected by ARPES, on the contrary, monolayer model fits it very well.

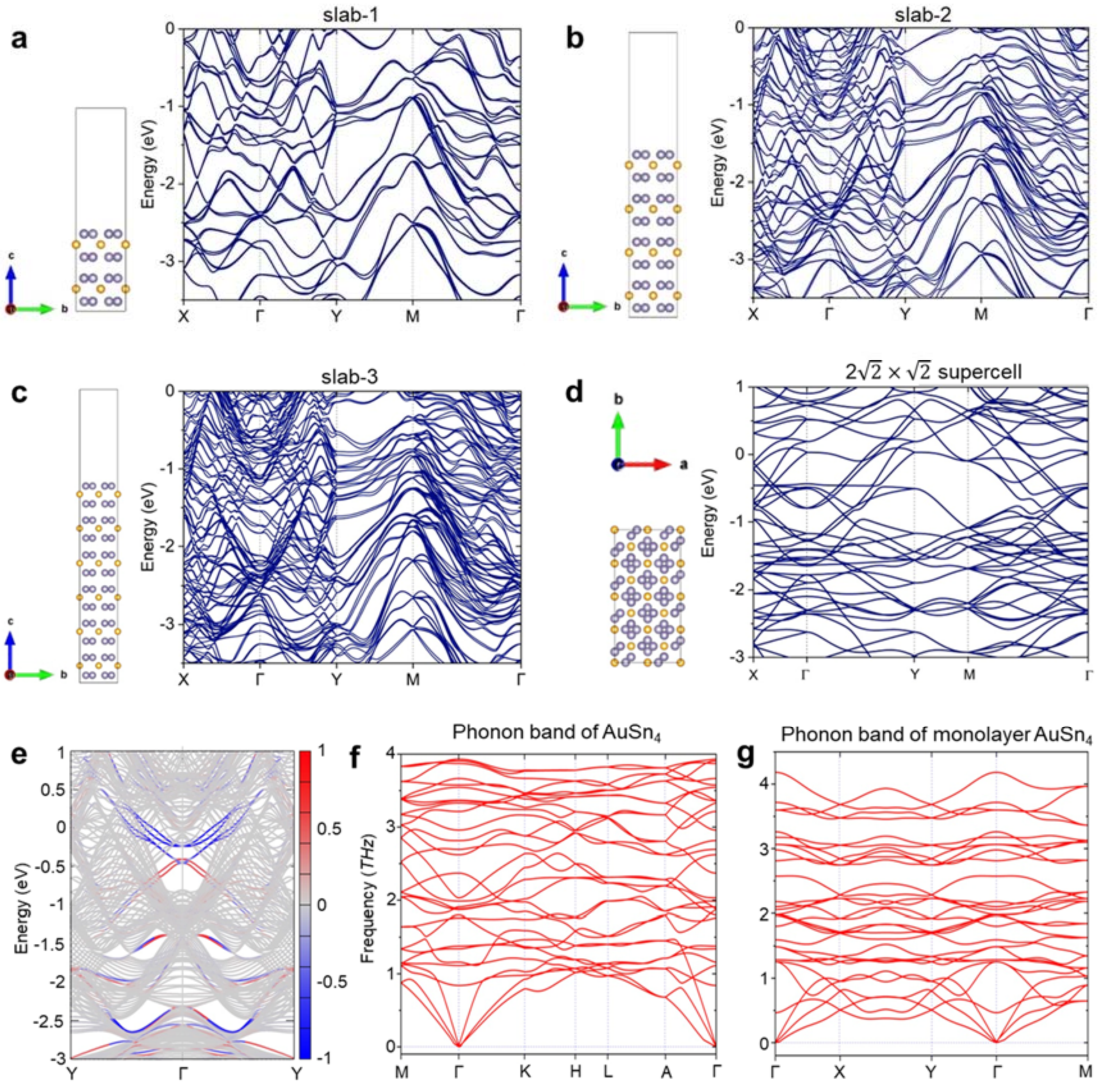

Supplementary Fig. S20 | Band structure for slabs with different thickness and phonon spectrum. **a**, n=2; **b**, n=4; **c**, n=6; **e**, n=12, where n is the number of layers. **d**, slab calculations that incorporate the $2\sqrt{2} \times \sqrt{2}$ superstructure. **f** and **g** are the phonon spectrum of bulk and monolayer $AuSn_4$, respectively.

Note 18. The analysis of the size of the vortex lattice and the vortex core radius.

Using the formula of $\xi_{ab} = \xi_c H_{c2}^{ab}/H_{c2}^{c}$, we calculated the value of the vortex core radius $r_c \approx \xi_{ab}$ to be ~ 190.8 nm[8,9]. Here, H_{c2}^{ab} and H_{c2}^{c} are the upper critical field for $H \parallel ab$ and $H \parallel c$, respectively, and ξ_{ab} and ξ_c are the coherence length for $H \parallel ab$ and $H \parallel c$, respectively. For $AuSn_4$, H_{c2}^{ab} = 1621Oe, H_{c2}^{c} = 643 Oe, and ξ_c = 71.69 nm. The distance between the center of the vortex lattice should be less than 410.73 nm estimated with the formula of $a = \sqrt{\frac{\phi_0}{B}}d$[10-12]. Here, d is a constant coefficient and B is the lower critical field. For $AuSn_4$, d = 1 and B = 118 Oe. We find that r_c is roughly comparable with $a/2$. Therefore, the reason for the absence of vortices in a superconductor, which may due to the overlap of vortex[13].

References

1. Mun, E., Ko, H., Miller, G. J., Samolyuk, G. D., Bud'ko, S. L. & Canfield, P. C. Magnetic field effects on transport properties of $PtSn_4$, *Phys. Rev. B* **85**, 035135 (2012).

2. Xu, C. Q. Enhanced electron correlations in the binary stannide $PdSn_4$: A homologue of the Dirac nodal arc semimetal $PtSn_4$, *Phys. Rev. Mat.* **1**, 064201 (2017).

3. Gurevich, A. Enhancement of the upper critical field by nonmagnetic impurities in dirty two-gap superconductors, *Phys. Rev. B* **67**, 184515 (2003).

4. Yonezawa, S. et al. Thermodynamic evidence for nematic superconductivity in $Cu_xBi_2Se_3$. *Nat. Phys.* **13**, 123–126 (2017).

5. Sigrist, M. & Ueda, K. Phenomenological theory of unconventional superconductivity, *Rev. Mod. Phys.* **63**, 239 (1991).

6. Mirhosseini, H. & Henk, J. *et al,* Unconventional spin topology in surface alloys with Rashba-type spin splitting. *Phys. Rev. B* **79**, 245428 (2009).

7. Song, R., Hao, N. & Zhang, P. Giant inverse Rashba-Edelstein effect: Application to monolayer $OsBi_2$. *Phys. Rev. B* **104**, 115433 (2021).

8. Chen, D. Y. *et al*. Superconducting properties in a candidate topological nodal line semimetal $SnTaS_2$ with a centrosymmetric crystal structure. *Phys. Rev. B* **100**, 064516 (2019).

9. Brandt, E. H. Vortex-vortex interaction in thin superconducting films. *Phys. Rev. B* **79**,134526 (2009).

10. Abrikosov, A. A. An influence of the size on the critical field for type II superconductors. *Dokl. Akad. Nauk.* **86**, 489 (1952).

11. Zavaritskii, N. Superconducting properties of thallium and tin films condensed at low temperatures. *Dokl. Acd. Nauk.* **86**, 501 (1952).

12. Abrikosov, A. A. The magnetic properties of superconducting alloys. *J. P*hys. *C*hem. *S*olids **2**, 199 (1957).

13. Tinkham, M. Introduction to superconductivity. Courier Corporation, (2004).
